# Renal cell cancer among African Americans: an epidemiologic review

**DOI:** 10.1186/1471-2407-11-133

**Published:** 2011-04-12

**Authors:** Loren Lipworth, Robert E Tarone, Joseph K McLaughlin

**Affiliations:** 1 International Epidemiology Institute, 1455 Research Boulevard, Suite 550, Rockville, MD 20850; 2 Department of Medicine, Vanderbilt University Medical Center and Vanderbilt-Ingram Cancer Center, Nashville, TN, USA

## Abstract

Incidence rates for renal cell cancer, which accounts for 85% of kidney cancers, have been rising more rapidly among blacks than whites, almost entirely accounted for by an excess of localized disease. This excess dates back to the 1970s, despite less access among blacks to imaging procedures in the past. In contrast, mortality rates for this cancer have been virtually identical among blacks and whites since the early 1990s, despite the fact that nephrectomy rates, regardless of stage, are lower among blacks than among whites. These observations suggest that renal cell cancer may be a less aggressive tumor in blacks. We have reviewed the epidemiology of renal cell cancer, with emphasis on factors which may potentially play a role in the observed differences in incidence and mortality patterns of renal cell cancer among blacks and whites. To date, the factors most consistently, albeit modestly, associated with increased renal cell cancer risk in epidemiologic studies among whites - obesity, hypertension, cigarette smoking - likely account for less than half of these cancers, and there is virtually no epidemiologic evidence in the literature pertaining to their association with renal cell cancer among blacks. There is a long overdue need for detailed etiologic cohort and case-control studies of renal cell cancer among blacks, as they now represent the population at highest risk in the United States. In particular, investigation of the influence on renal cell cancer development of hypertension and chronic kidney disease, both of which occur substantially more frequently among blacks, is warranted, as well as investigations into the biology and natural history of this cancer among blacks.

## Background

An estimated 58,240 new cases of kidney cancer and 13,040 deaths are expected in the United States in 2010, accounting for approximately 4% of all new primary cancer cases diagnosed [1]. Approximately 85% of kidney cancers are renal parenchyma (renal cell) cancers, while the remainder are mainly urothelial cancers of the renal pelvis [1]. Both renal cell and renal pelvis cancers are about twice as common among men as among women [1,2], with the mean age at diagnosis in the early 60s for renal cell cancer and in the late 60s for renal pelvis cancer. Epidemiologic characteristics and risk factors for renal pelvis cancer closely parallel for those for bladder cancer and have been addressed by the authors elsewhere [3].

Over the past several decades, incidence rates for renal cell cancer have been rising steadily each year in the United States [4]. Improved imaging technology has led to earlier detection and a decrease in the size of diagnosed renal cell tumors over time [5,6], but an increase in the incidence of large and late-stage renal cell cancers has also been observed [4,6,7]. A recent report showed that, while the rate for all cancers combined dropped 1.8% among men and 0.5% among women in the United States between 2001 and 2005, kidney cancer incidence is rising 1.7% per year for males and 2.2% per year for females [8].

**Figure 1:**
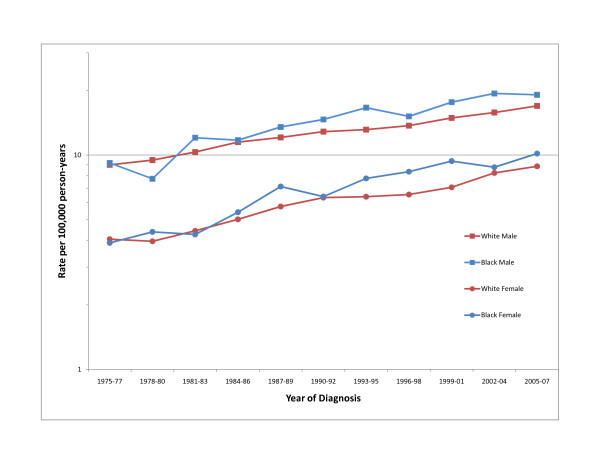
**Trends in age-adjusted (2000 United States standard) incidence of renal cell cancer by race and sex, 1974-2007 (Based on SEER data for nine geographic regions of the United States: Atlanta, Georgia; Connecticut; Detroit, Michigan; Hawaii; Iowa; New Mexico; San Francisco/Oakland, California; Seattle/Puget Sound, Washington; and Utah) **[2].

Perhaps the most prominent feature of the descriptive epidemiology of renal cell cancer has been the more rapid increase in incidence among blacks than whites, leading to a shift in excess from among whites to among blacks beginning in the mid-1980s [7] (Figure 1). By 2002-2007, the age-adjusted incidence rates of renal cell cancer among black men, white men, black women, and white women were 20.0, 17.4, 9.6 and 8.8 per 100,000 person-years, respectively [2]. The higher incidence of renal cell cancer among blacks is almost entirely accounted for by an excess of localized disease among patients of all ages and particularly among black men, which dates back to the 1970s [9,10]; for the periods 1973-77, 1978-82 and 1983-87, the incidence rates (per 100,000) for localized renal cell cancer were 3.7, 4.7 and 6.1 for black men, 3.5, 3.9, and 5.0 for white men, 1.8, 2.2 and 2.8 for black women, and 1.7, 1.8 and 2.3 for white women, respectively. Localized renal cell cancer has also been increasing at a significantly faster pace among blacks of all ages than among whites since the 1970s [10].

Early stage renal cell cancer is typically detected incidentally by imaging modalities which, on the basis of socioeconomic factors and accessibility to medical care, are unlikely to be utilized more frequently by blacks than whites [10,11]. Viewed historically, access to imaging and other medical technologies was not as available to blacks as it was to whites 30 to 40 years ago when the accelerated increase in the incidence of localized disease began among blacks. It is possible that the diagnostic work-up for co-morbidities more common among blacks may yield incidental findings of localized renal cell tumors. Presently, the higher prevalence of advanced chronic kidney disease among blacks [12] may result in increased renal surveillance with a concomitant increased detection of early renal tumors; but the most common co-morbidities among blacks, hypertension and diabetes, are not generally associated with increased imaging. Thus, the observed differences between blacks and whites in early stage renal cell tumor distribution dating back to the 1970s is unlikely to be a result of long-term greater access to and utilization of imaging technologies by blacks.

**Figure 2:**
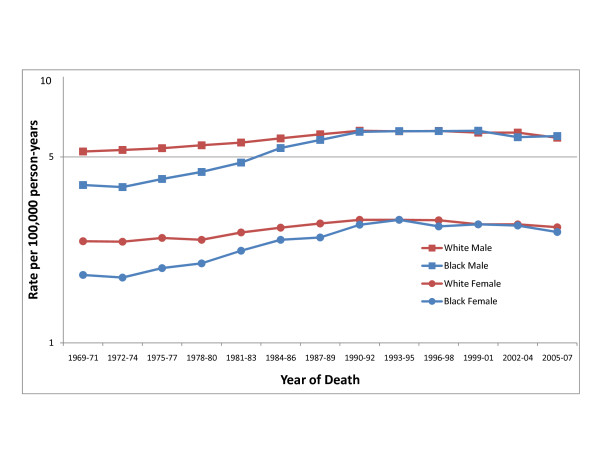
**Trends in age-adjusted (2000 United States standard) mortality from kidney cancer by race and sex, 1971-2007 (Based on National Center for Health Statistics data for the entire United States) **[2].

In contrast to these incidence trends, United States kidney cancer mortality rates, which include renal pelvis cancer but primarily reflect patterns related to renal cell cancer, have been virtually identical among blacks and whites for both men and women since the early 1990s (Figure 2). This equivalence in mortality despite higher incidence does not appear to be a result of higher rates of nephrectomies among blacks [9]; rates of nephrectomy are in fact lower among blacks than among whites, while cause-specific survival is comparable or higher among blacks.

These puzzling incidence and mortality trends suggest that the biology of renal cell cancer may differ between blacks and whites. In particular, renal cell cancer may be a less aggressive tumor among blacks [9], as suggested by the favorable stage distribution among blacks and their higher survival, particularly for distant and unstaged cancer. Zini et al. [13] have also reported similar survival despite lower nephrectomy rates among blacks, although others have reported lower survival rates among blacks compared with whites with renal cell cancer [14,15].

## Methods

We have conducted a review of epidemiologic studies of risk factors for renal cell cancer, identified through a PubMed search of the literature. For the purposes of this review, all papers published through 2010 were initially identified by use of either the term "renal cancer" or the term "kidney cancer" together with the term "risk factor" or "epidemiology." Moreover, all review papers addressing risk factors for kidney cancer in general or renal cell cancer in particular were identified, and references were examined to supplement, if necessary, papers identified through the initial search.

A qualitative evaluation and summary of the results of individual studies is presented; rather than citing every paper we have identified, we have chosen to emphasize those results that reflect consistency in the literature and, more importantly, that may potentially play a role in the observed differences in incidence and mortality patterns of renal cell cancer among blacks and whites. No consistent association has been demonstrated between renal cell cancer and social class variables such as education or income. Despite considerable racial differences in the descriptive epidemiology of this malignancy, there have been virtually no etiologic epidemiologic studies presenting results for lifestyle and other risk factors for renal cell cancer separately among blacks, and even the small number of multiethnic studies conducted to date have generally not presented results separately for blacks and whites [16-20], often because of small numbers of blacks in the study population.

## Results and Discussion

### Genetic susceptibility

Renal cell cancer occurs in both hereditary and sporadic forms. Hereditary renal cell cancer tends to occur earlier in life than sporadic forms of the disease, and often involves bilateral, multifocal tumors [21]. Having a first degree relative with kidney cancer has been associated with a 2- to 5-fold increased risk [22]. A recent meta-analysis suggested that the observed risk is higher when the affected relative is a sibling rather than a parent, supporting the role of recessive, low-penetrance genes in familial renal cell cancer [22,23]. In a case-control study that evaluated whites and blacks separately and relied on self-reported family history of kidney cancer, the relative risk for renal cell cancer associated with a family history of kidney cancer was higher, although not statistically significantly so, among blacks than among whites [24]; for any first-degree relative with kidney cancer, the relative risk for renal cell cancer was 3.96 (95% CI 1.45-10.84) among blacks and 1.98 (95% CI 0.99-4.03) among whites.

Although fewer than 5% of renal cell cancers are explained by inherited predisposition [21,25], no other cancer has as many different types of genetic predisposition as renal cell cancer [26]. Renal medullary cancer is a rare, usually fatal primary kidney tumor that afflicts almost exclusively young blacks with sickle cell trait or disease [27]. The tumor, which displays unique clinical and pathological characteristics, originates from the epithelium of renal collecting ducts, and is almost always metastatic at the time of diagnosis, with death usually occurring within a few months of diagnosis. This highly aggressive tumor was first described in 1995 [28], and approximately 120 cases have been reported to date, virtually all among patients aged less than 40 years [29].

Other rare genetic forms of renal cell cancer are von Hippel-Lindau (VHL) disease (predisposing to clear cell cancer), hereditary papillary renal cell carcinoma (HPRC), hereditary leiomyomatosis renal cell cancer (papillary), Birt-Hogg-Dubé syndrome (mainly chromophobe and oncocytoma), chromosome 3 translocation-associated (clear cell), tuberous sclerosis (clear cell), mutated succinate dehydrogenase genes, and Cowden syndrome [21,25,30]. Genes have been identified related to these inherited forms of renal cell cancer: the tumor suppressor *VHL *gene located on the distal region of chromosome 3p [31], the *EPAS-1 *gene encoding the HIF-2α transcription factor that is critical in kidney carcinogenesis for VHL-deficient RCC [32], six different rare germline translocations affecting chromosome 3 in a small number of families with familial clear cell renal cancer [33-35], the *MET *gene for hereditary papillary carcinoma, the *FLCN *gene for Birt-Hogg-Dubé syndrome, the *FH *gene for hereditary leiomyomatosis renal cell cancer, three succinate dehydrogenase genes including *SDHB *[36], and the tumor suppressor phosphatase and tensin homolog (*PTEN*) and *KILLIN *genes in Cowden syndrome [37]. All of the identified genes are involved in pathways that respond to metabolic stress or nutrient stimulation [36]. Aside from the sickle cell trait, none of the inherited syndromes associated with increased risk of kidney cancer has been reported to have a higher prevalence in blacks.

### Chronic kidney disease

It is well-established that patients on renal replacement therapy for end stage chronic kidney disease are at increased risk for many cancers [38]. In particular, several large studies conducted in North America, Europe, or Australia and New Zealand have reported substantially elevated relative risks for renal cell cancer among patients on dialysis or renal transplantation therapy for end-stage kidney disease, independent of underlying primary renal disease, with relative risks ranging from 3.3 up to almost 15 [39-44]. The risk for renal cell cancer rises with duration of dialysis treatment [44]. Acquired renal cystic disease of the native kidneys, which occurs among dialysis patients independent of age or primary renal disease and whose prevalence also increases with duration of dialysis, is believed to account for the excess of renal cell carcinoma among patients on dialysis [44,45]. The increased cancer risk among transplant recipients is likely due, at least in part, to the use of immune-suppressing medications [38]. Evidence for an association between early stage chronic kidney disease and renal cell cancer risk is more limited [42,46].

Rates of end stage renal disease, in particular hypertension- and diabetes- related end stage renal disease, are consistently reported to be at least four-fold higher among blacks than among whites [47-49]. The rate of hypertension-related end stage renal disease is 20 times higher among 20 to 44 year old black males compared with their white counterparts [48]. In contrast, rates of earlier stage chronic kidney disease are similar or increased among whites compared with blacks [12,50,51]. Between 1999 and 2004, the prevalence of all stages of chronic kidney disease combined, defined by a combination of glomerular filtration rate (GFR) < 60 ml/min/1.73 m^2 ^and high proteinuria, was reported to be 16.1% among whites and 19.9% among blacks [52]. Detailed analyses based on GFR measurements have demonstrated a white excess for mild chronic kidney disease giving way to a black excess for moderate to severe chronic kidney disease [52,53]. For example, in the Reasons for Geographic And Racial Differences in Stroke (REGARDS) cohort study, the black:white odds ratio for impaired kidney function was 0.74 (95% CI 0.66-0.84) among individuals with a GFR between 50 and 59 ml/min/1.73 m^2^, increasing to 2.96 (95% CI 1.72-5.11) among those with a GFR between 10 and 19 ml/min/1.73 m^2 ^[53]. It has also been suggested that a lower level of blood pressure may be necessary to slow the decline in renal function among blacks with chronic kidney disease and hypertension compared with whites [54,55], but racial differences in the susceptibility to renal damage from elevated blood pressure have been reported to persist even after adjustment for differences between blacks and whites in hypertension and hypertension-control [56].

A recent study [57] showed that, among blacks, focal segmental glomerulosclerosis and hypertension-attributed end-stage kidney disease are strongly associated with variants in the *APOL1 *gene on chromosome 22, supporting a genetic basis for predisposition to kidney disease in blacks. The *APOL1 *risk alleles for renal disease occur in more than 30% of African-American chromosomes but appear to be absent in whites [57], and it is possible that a similar genetic marker associated with renal cell cancer may exist among blacks.

### Obesity

Regardless of study design, renal cell cancer is consistently associated with obesity. The association of renal cell carcinoma with obesity has been reported in virtually all epidemiologic studies, including many large prospective studies conducted in various populations, and most studies have observed an effect of elevated BMI among both men and women. Only one large cohort study of 3,668,486 white and 832,214 black male US veterans has specifically evaluated obesity-related renal cell cancer risk separately among blacks, but it used a hospital diagnosis of obesity rather than BMI as the measure of exposure [58]. The relative risks for renal cell cancer associated with clinical obesity were 1.74 (95% CI 1.58-1.90) among white men and 1.38 (95% CI 1.09-1.74) among black men. Risk for renal cell cancer was significantly elevated among white men with or without hypertension, while among black men risk of renal cell cancer was not elevated among those without hypertension [58]. In a large, Swedish population-based study that used measurements of height and weight rather than questionnaire-derived data, the relative risks for renal cell cancer associated with being overweight (BMI 25.0-29.9) or obese (BMI > 30) were 1.28 and 1.82, respectively [59].

Recent quantitative summary analyses of the epidemiologic evidence reported that associations with increased BMI were slightly stronger in women than in men [60-62]; Renehan et al. presented summary risk ratios of 1.24 (95% CI 1.15-1.34) among men and 1.34 (95% CI 1.25-1.43) among women per 5 kg/m^2 ^increase in BMI [62], while pooled relative risks from cohort studies were 1.06 among women and 1.05 among men per unit increase in BMI [60,61].

The rising incidence of renal cell cancer is likely to be accounted for in part by the increasing prevalence of obesity [63,64], although increases in obesity prevalence observed since the 1980s may not be continuing at the same rate in recent years, particularly among white men [62]. In 2007-2008 in the United States, the prevalence of obesity (BMI ≥ 30) and of obesity and overweight combined (BMI ≥ 25) were 33.8% and 68%, respectively [62]. The prevalence of obesity in the United States is up to 50% higher among blacks when compared with whites, with the difference more pronounced among women than men [62,63]. The proportion of renal cell cancer attributable to being overweight and obese could be as high as 40% in the United States and Canada [64-66].

Several credible mechanisms have been suggested for the association between obesity and renal cell cancer. Obesity regulates the release of free fatty acids and adipose tissue-derived hormones and cytokines, including leptin and adiponectin [34]. While levels of most cytokines, including leptin, are increased in obese individuals, serum adiponectin levels are inversely correlated with BMI among both blacks and whites [67-69], and appear to be lower in blacks compared with whites [67,69-74]. A few small studies have shown reduced adiponectin levels in patients with renal cell cancer, and an inverse correlation between adiponectin levels and markers of tumor aggressiveness, including tumor size and metastasis [75-77]. Moreover, circulating levels of adiponectin are inversely associated with insulin resistance, and elevated circulating insulin levels and increases in the bioavailability of insulin-like growth factor-I (IGF-I) associated with obesity could in turn lead to increased cell proliferation and the development of renal cell cancer [78-80]. A recent study showed that, while BMI was associated with IGF-1 levels regardless of race, black women had higher mean IGF-1 levels compared with white women adjusted for BMI. Lipid peroxidation of proximal renal tubules, which is increased among obese subjects, has also been hypothesized to play a role in the association of obesity with renal cell cancer through the formation of DNA adducts [81].

### Hypertension

While it is difficult to separate the effects of hypertension or its treatment on renal cell cancer risk, as they are highly correlated variables, the collective epidemiologic evidence to date suggests that it is hypertension itself that plays a role in the etiology of this tumor. There appears to be no excess risk for renal cell cancer associated with use of any type of class of antihypertensive medications or with diuretic use, after adjustment for high blood pressure [82-86]. Most studies have reported relative risks for renal cell cancer associated with either recorded blood pressure or reported hypertension ranging between 1.2 and 2 or greater, and several cohort studies have demonstrated an increased risk even after exclusion of the first few years of follow-up, when early stage, prediagnostic renal tumors may themselves lead to elevated blood pressure [20,82-84,87,88].

Trends of increasing renal cell cancer incidence may be associated with the increasing prevalence of hypertension in the United States [89-91], particularly among blacks. While awareness and treatment of hypertension has improved among blacks over time, a substantially higher prevalence of hypertension among blacks compared with whites persists, as does the large difference between blacks and whites in the proportion of patients with hypertension who are receiving treatment [91,92]. The diagnostic work-up for hypertension, while more common among blacks, is not generally associated with increased imaging, so hypertension is unlikely to account for the more common occurrence of incidental localized renal cell tumors among blacks compared with whites. In fact, detection bias due to incidental diagnosis during work-ups for hypertension was not supported in a study which directly evaluated that hypothesis [93]. Although the effect of hypertension on renal cell cancer is believed to be independent of obesity, adiponectin levels are lower among blacks than among whites, and low adiponectin levels have been linked in a few studies to hypertension as well as obesity. Moreover, despite the linear relationship between increased body weight and increased blood pressure in whites, it has been suggested that blacks have higher blood pressures at lower weights compared with whites [94].

Several biologic mechanisms for the association between high blood pressure and renal cell cancer risk have been proposed, including hypertension-induced renal injury or metabolic or functional changes within the renal tubule induced by hypertension increasing susceptibility to carcinogens. It has also been speculated that elevated levels of insulin-like growth factor-I (IGF-I) or lipid peroxidation associated with hypertension, as well as up-regulation of hypoxia-inducible factors, could contribute to the development of renal cell cancer.

### Cigarette smoking

Cigarette smoking is a recognized though moderate cause of renal cell cancer [95]. A meta-analysis [96] of data from 19 case-control studies (8,032 cases and 13,800 controls) and 5 cohort studies (1,457,754 participants with 1,326 renal cell cancer cases) reported statistically significant relative risks of 1.5 and 1.2 for male and female smokers, respectively. There was a strong dose-dependent increase in risk, up to 2- and 1.6-fold among heavy (21 or more cigarettes per day) men and women smokers, respectively. There was a significant decline in risk in both sexes with years of cessation, with a 15 to 30% reduction in risk 10 to 15 years after quitting [96]. Approximately 20 to 30% of renal cell cancers among men and 10 to 20% among women are estimated to be attributable to cigarette smoking [89,97,98]. According to the 1998 Surgeon General's report on tobacco use, the prevalence of smoking declined among African Americans between 1978 and 1995, from 37.3% to 26.5% overall, and while blacks have higher rates of smoking they are less likely than whites to be heavy smokers and smoke fewer cigarettes per day, so this causal factor is an unlikely explanation for the excess incidence of renal cell cancer among blacks [99].

### Statins

Statins are widely used drugs for the treatment of lipid disorders, particularly hypercholesterolemia. Anti-tumorigenic properties of statins have been documented, including their inhibition of proliferation and promotion of apoptosis, but their potential effectiveness for the primary prevention of cancer has not been conclusively demonstrated. Despite observed differences between blacks and whites in both the prevalence of hypercholesterolemia and its treatment with statins, both lower among blacks than whites [100,101], to our knowledge no study has evaluated the association between statin use and renal cell cancer among blacks.

### Other lifestyle factors

Several other factors have been extensively evaluated in epidemiologic studies of renal cell cancer. However, credible or consistent associations of these factors with renal cell cancer have not been reported, and they do not play an obvious role in explaining the unusual patterns of renal cell cancer incidence and mortality among blacks and whites. The collective epidemiologic evidence related to these characteristics and risk factors has been addressed by the authors in detail elsewhere [102,103] and, therefore, will be mentioned here only in brief.

#### Analgesics

Historically, the causal connection of heavy use and abuse of phenacetin-containing analgesics and transitional cell cancers of the renal pelvis has long been recognized [3]. Phenacetin's effect on adenocarcinomas of the renal parenchyma, however, is inconclusive, and it is now impossible to assess because phenacetin-containing analgesics have been off the market for up to 30 years in most countries and reliable recall of past intake is no longer achievable. Neither acetaminophen, the major metabolite of phenacetin, nor aspirin has been credibly associated with an increase in renal cell cancer risk. To date, there is no epidemiologic investigation of analgesic use and renal cell cancer among blacks.

#### Diet

Although originally thought almost 40 years ago to play a key role in renal cell cancer etiology, dietary factors have not fulfilled their early promise, as renal cell cancer has not been convincingly linked to any specific dietary factor. To our knowledge, there has been no etiologic study of renal cell cancer and diet to publish results specifically among blacks. A protective effect for overall fruit and vegetable consumption has been generally accepted, but two recent large prospective studies, one based on 375,851 participants in the European Prospective Investigation into Cancer And Nutrition (EPIC) [104] and the other based on 120,852 men and women in the Netherlands cohort study on diet and cancer (NLCS) [105], reported no protective effect of vegetable and/or fruit consumption on renal cell cancer. Some epidemiologic studies suggest that elevated protein consumption may be a risk factor for renal cell cancer. There may be some biologic plausibility to a high protein diet affecting risk of renal cell cancer, because animal studies have shown protein intake can induce renal tubular hypertrophy, but the large international renal cell cancer case-control study failed to provide clear support for this hypothesis [106], as did a large, pooled analysis of 13 cohort studies [107].

#### Alcohol consumption

Early ecologic studies consistently suggested a positive correlation between kidney cancer and per capita consumption of alcohol, but these ecologic findings were not confirmed by numerous analytic epidemiologic studies of renal cell cancer conducted during the ensuing two decades [100,101]. After adjustment for the confounding effect of cigarette use, virtually all studies, including cohort studies of alcoholics and brewery workers, showed no association between alcohol consumption and renal cell cancer.

By contrast, a post hoc hypothesis has recently appeared in the literature that moderate levels of alcohol consumption may be protective for renal cell cancer. The findings of recent individual studies show considerable heterogeneity and inconsistency with respect to the categories of alcohol consumption, the amount of alcohol intake reportedly associated with decreased renal cell cancer risk, and differential observations between men and women. A pooled analysis of data from 12 prospective studies of renal cell cancer was recently published, based on results of five published studies as well as numerous others which had not previously published their data related to alcohol consumption [108]. The pooled analysis was based on 1430 incident cases of renal cell cancer (719 men and 711 women), and demonstrated an apparent inverse-response relation at levels of consumption equivalent to less than a drink per day, with no further protective effect at levels of intake above a drink a day. It is difficult to imagine the biologic mechanism that could explain this unusual type of dose-response pattern. Unless of course one invokes a hormesis-like effect of low-dose alcohol on renal cancer risk while other body organs apparently do not enjoy this low-dose anti-carcinogenic effect.

Finally, alcohol itself is a known human carcinogen and heavy alcohol drinking has been conclusively linked to increased risks of numerous types of cancer, including oral, pharyngeal, laryngeal, esophageal, liver and probably breast and colon and rectum [109]. A protective effect of alcohol consumption on renal cell cancer at very low levels of intake has little biologic plausibility or credibility. In addition to the extensive analytic epidemiologic evidence from the past 40 years, the descriptive patterns of renal cell cancer are not consistent with an inverse association with alcohol intake. In particular, the rate of renal cell cancer among men is twice that among women worldwide, whereas men tend to consume alcohol at substantially higher levels than women.

#### Hormonal and reproductive factors

Reductions in risk of renal cell cancer have been reported among users of oral contraceptives in some [110,111] but not all studies [112,113], and in the large international case-control study protection was restricted to non-smokers [110]. Hormones have induced renal tumors in laboratory animals; however, with the exception of an almost two-fold increased risk among women with high parity compared with nulliparous women in some studies, after adjustment for obesity [110,111,114], evidence for a role of hormonal or reproductive factors in the etiology of renal cell cancer in humans is not strong or consistent.

### Occupation

As compared with bladder cancer, renal cell cancer has not been convincingly linked to any occupational exposure. However, because of the large number of epidemiologic studies of this cancer, particularly case-control studies, that have been conducted over the last three decades, a number of sporadic associations have been reported between exposures or jobs/industries and renal cell cancer. Most attention has been focused on asbestos, gasoline and, more recently, the solvent trichloroethylene (TCE). Extensive reviews and meta-analyses of occupational cohort studies have failed to confirm suspicions of increased risk of kidney cancer among workers exposed to asbestos or gasoline [115-117].

Three epidemiologic studies conducted in one area of Germany, which were initiated in response to a cluster of renal cell cancer cases observed in a plant, reported strikingly elevated relative risks for renal cell cancer associated with TCE exposure [118-120]. The findings contrast starkly with results from other investigations, and several serious methodological shortcomings of these studies have been noted [121-123] limiting any conclusion that can be drawn. To date, seven occupational cohort studies have evaluated the relationship between TCE exposure and specific types of cancer. The two largest, which both employed sophisticated methods of exposure assessment and internal and external comparisons [124,125], reported no significantly increased risk of renal cell cancer among TCE exposed workers. The most recent cohort study [126], conducted in Denmark, evaluated cancer incidence among 40,049 workers with presumed TCE exposure and found a weak association with renal cell cancer among those thought to be heavily exposed to TCE. The weight of the evidence to date, however, does not provide consistent, credible support for the hypothesis that TCE is a cause of renal cell cancer in humans [127]. Whether TCE is a renal carcinogen in humans remains an open question, which will require more and better research.

## Conclusions

To date, the factors most consistently associated with increased renal cell cancer risk in epidemiologic studies - obesity, hypertension, cigarette smoking - likely account for less than half of these cancers among whites, and there is scant published evidence pertaining to their association among blacks with renal cell cancer. In light of the enigmatic differences in incidence and mortality trends in renal cell cancer among blacks, there is an urgent need for detailed etiologic cohort and case-control studies of renal cell cancer among blacks, as they now represent the population at highest risk in the United States. In particular, detailed investigations are needed of the influence on renal cell cancer development of hypertension and chronic kidney disease, both of which occur much more commonly among blacks, as well as investigations of genetic markers which may reflect greater susceptibility to renal cell cancer among blacks. Further, detailed studies of the biology and natural history of renal cell cancer among blacks are essential.

## Declaration of competing interests

The authors declare that they have no competing interests.

## Authors' contributions

LL, RET and JKM all made substantial contributions to conception, analysis and interpretation of published results for this review, and drafting of the manuscript. All authors have given final approval of the version to be published.

## Pre-publication history

The pre-publication history for this paper can be accessed here:

http://www.biomedcentral.com/1471-2407/11/133/prepub
